# Early experience with proton craniospinal irradiation in adult patients with leptomeningeal disease

**DOI:** 10.1186/s13014-025-02618-7

**Published:** 2025-04-22

**Authors:** Felix Ehret, Ammy M. Yuan, Ariel E. Marciscano, Stephen Zieminski, Peggy A. Leland, Melin J. Khandekar, Kevin S. Oh, Helen A. Shih

**Affiliations:** 1 Department of Radiation Oncology, Massachusetts General Hospital, Harvard Medical School, Boston, MA USA; 2 Charité – Universitätsmedizin Berlin, Corporate Member of Freie Universität Berlin and Humboldt-Universität zu Berlin, Department of Radiation Oncology, Berlin, Germany; 3 German Cancer Consortium (DKTK), partner site Berlin, a partnership between DKFZ and Charité – Universitätsmedizin Berlin, Berlin, Germany; 4 Harvard University, Cambridge, MA USA; 5 Massachusetts General Hospital, Boston, MA USA

**Keywords:** Leptomeningeal disease, Leptomeningeal metastasis, Brain metastasis, Proton therapy, Craniospinal irradiation, CSI

## Abstract

**Background:**

Leptomeningeal disease (LMD) is a fatal complication of cancer linked to poor survival rates and limited treatment options. While photon involved-field radiotherapy is the standard of care for local palliation and symptom alleviation, it lacks durable disease control. Recent data suggest proton craniospinal irradiation (pCSI) to be a promising treatment option, potentially prolonging progression-free survival (PFS) and overall survival (OS). Herein, we report our initial experience with pCSI for treating LMD from solid malignancies.

**Methods:**

Adult patients treated with pCSI for LMD were identified, with analysis of patient, tumor, and treatment characteristics as well as clinical outcomes.

**Results:**

Nine patients were eligible for analysis who were treated between February 2023 and February 2024. The median age at pCSI and Karnofsky performance status (KPS) were 58.6 years and 80%, respectively. The primary disease was breast cancer in 33.3%, and LMD involved both the brain and spine in 55.5%. Approximately half of the patients (55.5%) had a cerebrospinal fluid diversion before treatment, and nearly all patients underwent pCSI with 30 Gy (relative biological effectiveness) in 10 fractions. All patients completed pCSI as planned. The median clinical and central nervous system (CNS) radiographic follow-up periods were both 3.5 months. Six deaths were observed during the available follow-up. The median PFS, CNS PFS, and OS were 2.7, 4.0, and 4.0 months, respectively. Younger age, higher KPS, and concurrent treatment with targeted therapy were associated with longer OS, while cases with LMD involving both the brain and spine had shorter survival. The observed toxicity was manageable, without any occurrence of grade 4 or 5 toxicity.

**Conclusion:**

pCSI can be an effective and safe treatment option for a highly selected population of patients with LMD. Further data and prospective studies are warranted to clarify its role in the management of LMD.

**Supplementary Information:**

The online version contains supplementary material available at 10.1186/s13014-025-02618-7.

## Introduction

Leptomeningeal disease (LMD), i.e., the dissemination of malignant cells into the cerebrospinal fluid (CSF)-filled leptomeningeal space surrounding the brain and spinal cord, is associated with significant morbidity and mortality [1]. Clinically detected in 5 to 10% of patients with solid tumors, LMD is often underdiagnosed. Up to 30% of all patients with malignancy and neurological symptoms are found to have LMD upon autopsy [2, 3]. Due to advancements in imaging techniques and systemic disease control, LMD cases are on the rise [3, 4]. Despite advances in care, prognosis remains dismal, with an overall survival (OS) of approximately 4 to 6 months from the time of diagnosis if treated, also depending on further factors, such as primary tumor, the burden of disease, presence of additional metastases, comorbidities, and performance status [1]. Without treatment, the OS is often shorter, ranging from weeks to a few months [1].

The current treatment for LMD is primarily palliative, as cure is not deemed feasible. Various treatment options exist. Photon involved-field radiotherapy (IFRT) is the standard of care for local palliation but often lacks durable disease control [5]. Systemic treatments, including intrathecal chemotherapy, represent another option. These treatments face challenges due to the blood-brain barrier and the diffuse nature of LMD, limiting their long-term effectiveness [2]. However, new drugs with better central nervous system (CNS) penetrance might provide new avenues for the effective use of systemic therapies [3].

Photon craniospinal irradiation, targeting the full neuroaxis and, therefore, the full spatial LMD spread, while often used in pediatric cancer patients, may cause considerable and potentially intolerable toxicity in adult patients both due to acute symptoms of nausea, vomiting, and significant myelosuppression [6]. Proton craniospinal irradiation (pCSI), leveraging the distinct dosimetric advantages of particle therapy, offers the possibility of delivering radiation to the neuroaxis in a much more conformal way [7].

Recent data from a randomized phase II trial comparing pCSI to IFRT in 63 patients with LMD from either breast cancer or non-small cell lung cancer (NSCLC) demonstrated superior CNS progression-free survival (PFS) and OS favoring pCSI without differences in serious adverse effects [8]. In addition, an exploratory registry arm of 35 patients with other solid tumors tolerated pCSI well. Other studies and case reports underlined the potential of this treatment approach [9–11]. Based on these encouraging results, this study aims to report our early institutional experience with pCSI for patients with LMD from solid malignancies.

## Materials and methods

This retrospective cohort study analyzed all patients undergoing pCSI for LMD from solid malignancies at our institution. Patients were eligible for evaluation if they had pathologically confirmed solid tumor malignancies with LMD established through imaging and/or CSF cytology. pCSI was done via spot scanning, with patients being immobilized in the supine position with a neutral head position on a carbon fiber board with custom head cushions. Beam angles were either posterior or posterior oblique. For target overlap organs at risk (OARs), the aim for maximum dose heterogeneity was under 102%. The dose constraints for OARs included 25 Gy (relative biological effectiveness (RBE)) maximum dose for eyes and cochleas, 10 Gy (RBE) maximum dose for lenses, and 31 Gy (RBE) maximum dose for the posterior scalp. The Astroid (.decimal, Sanford, FL, USA) treatment planning system was utilized for all patients. Image guidance was based on 2D kV imaging.

Descriptive statistics were used to summarize patient, tumor, and treatment characteristics. Continuous variables were described using medians, means, interquartile ranges (IQR), and minimum and maximum values. Categorical variables were summarized using absolute numbers and frequencies. Toxicity was graded according to the Common Terminology Criteria for Adverse Events (CTCAE) version 5.0 for anemia, thrombocytopenia, leukopenia, fatigue, headache, nausea, weight loss, muscle weakness, alopecia, anorexia, seizures, decreased vision, and blurred vision [12]. Due to the expected short survival, only acute toxicity, i.e., during or within 90 days upon completion of pCSI, was assessed. The last radiographic follow-up was defined as the last date patients received CNS imaging after pCSI treatment, including either magnetic resonance imaging (MRI) or computed tomography scans of the CNS. The last clinical follow-up was defined as the last date patients were seen in person at the hospital by a managing physician. The use of systemic therapies like chemotherapy, targeted therapy, and immunotherapy was considered concurrent during pCSI if these therapies were received within 60 days before, during, and 60 days after pCSI treatment. Systemic treatments were generally withheld during pCSI at the physician's discretion.

CNS progression was defined as the appearance of new or enlarging CNS lesions detected by available imaging. CNS PFS was considered the time from the start of pCSI to the first occurrence of CNS progression or death from any cause, whichever occurred first. PFS was defined as the time from the start of pCSI to the first occurrence of any disease progression or death from any cause, whichever occurred first. OS was the time from the first day of pCSI to death from any cause. Censoring for CNS PFS, PFS, and OS was done on the last available clinical follow-up for patients without observed events. Time-to-event analyses were performed using the Kaplan-Meier estimator. Median survival times and 95% confidence intervals (CI) were derived. The data analysis was performed with STATA MP 17.0 (StataCorp, College Station, TX, USA). Due to the anticipated low number of patients and events, no inferential statistics were conducted. This study was approved by the local institutional review board (protocol number 2024P000907). Studies on the use of pCSI for LMD in adult patients with solid tumors were collected through a non-systematic literature search using PubMed.

## Results

Nine patients met the inclusion and exclusion criteria and were treated between February 2023 and February 2024. The median age at pCSI was 58.6 years, with the most common primary disease being breast cancer (3/9, 33.3%), followed by lung cancer (2/9, 22.2%). Two breast cancer patients had triple negative disease. One patient with LMD from ovarian cancer showed a BRCA1 mutation. The patient suffering from metastatic NSCLC had an epidermal growth factor receptor (EGFR)-mutant tumor. The patient with gastric adenocarcinoma had a tumor expressing programmed cell death-ligand 1 (PD-L1) and showing a fibroblast growth factor receptor 2 (FGFR2) amplification.

The majority of patients were female (7/9, 77.7%). Regarding clinical status, the median Karnofsky Performance Status (KPS) at the time of pCSI was 80%. CSF diversion before pCSI was performed in five patients (55.5%). Four patients (44.4%) had received prior radiotherapy, with two patients (22.2%) receiving treatment for (non-CNS) primary disease and two (22.2%) having undergone prior CNS-directed radiotherapy for brain metastases. Both patients received multiple stereotactic radiosurgery courses for brain metastases. LMD diagnosis was made via imaging and CSF in four patients (44.4%), imaging alone in four patients (44.4%), and CSF alone in one patient (11.1%). Radiographic LMD spread was found in the brain in four patients (50.0%) and both the brain and spine in four patients as well (50.0%). Linear LMD was present in five patients (55.5%), both linear and nodular in three patients (33.3%), and CSF-only in one patient (11.1%). Six patients (66.6%) had active, i.e., uncontrolled, extra-CNS disease at the time of pCSI, while three patients (33.3%) had controlled disease. At the time of pCSI, three patients (33.3%) had parenchymal brain metastases, with the number of brain metastases ranging from 1 to 6. A significant portion of the cohort received concurrent systemic treatments. Specifically, 44.4% of patients (4 out of 9) received chemotherapy, 55.5% of patients (5 out of 9) received targeted therapies during this time frame, while 22.2% of patients (2 out of 9) received some form of immunotherapy in this period.

**Fig. 1:**
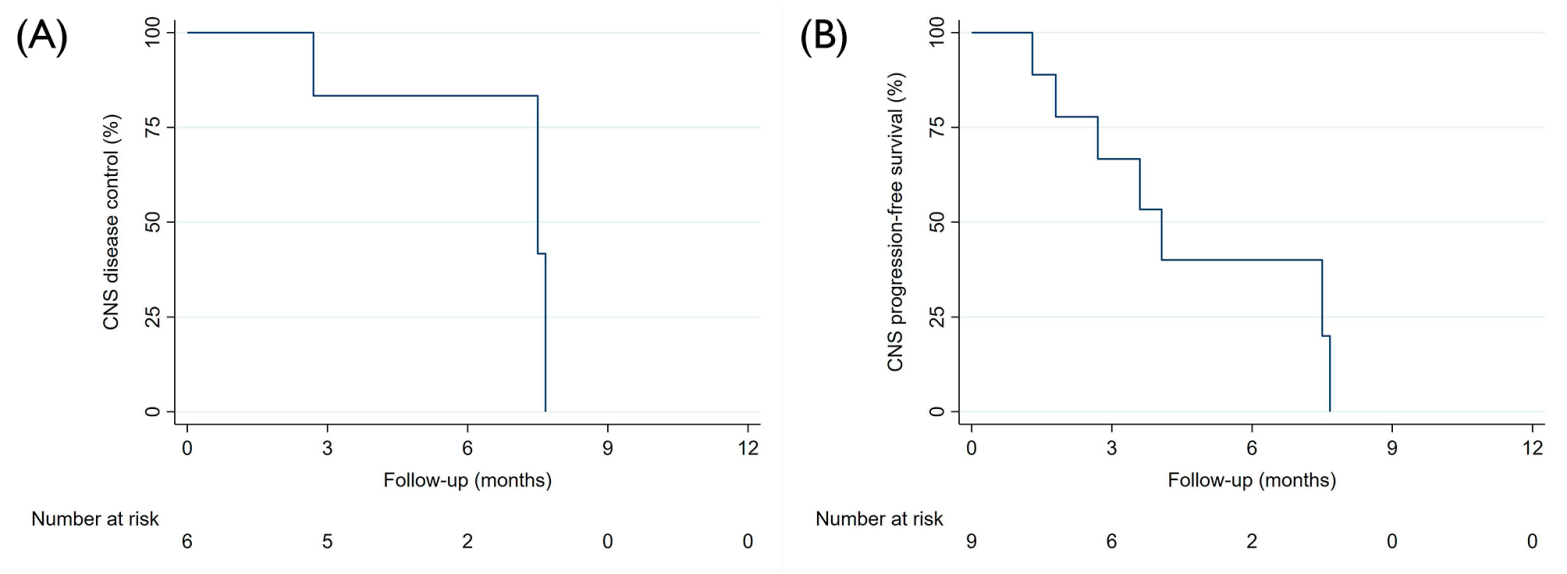
Time to (**A**) CNS progression, (**B**) CNS progression-free survival

**Fig. 2:**
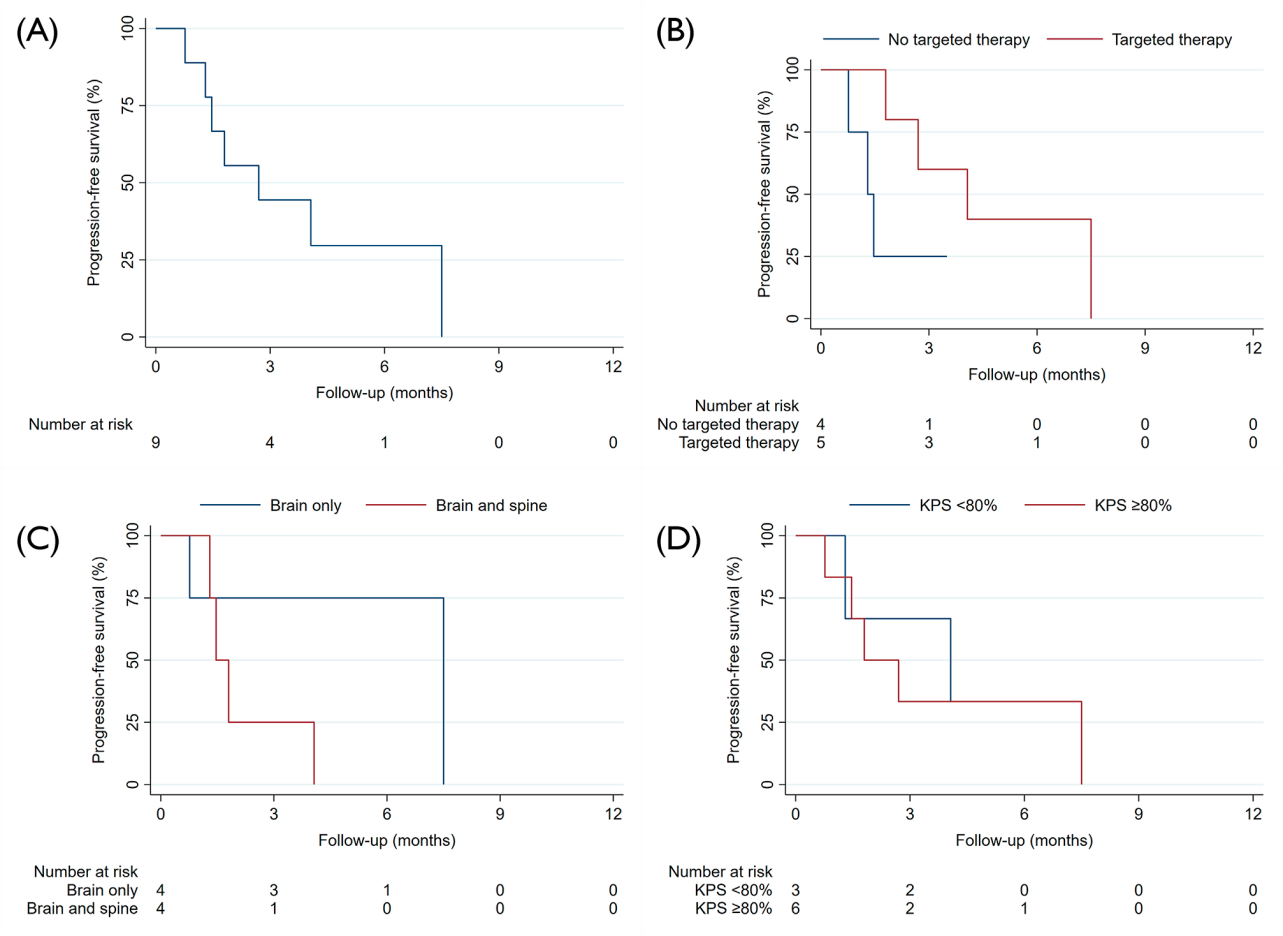
Progression-free survival for (**A**) Full cohort, (**B**) Stratified for concurrent targeted therapy, (**C**) Stratified for the extent of LMD, (**D**) Stratified for the median Karnofsky performance status

**Fig. 3:**
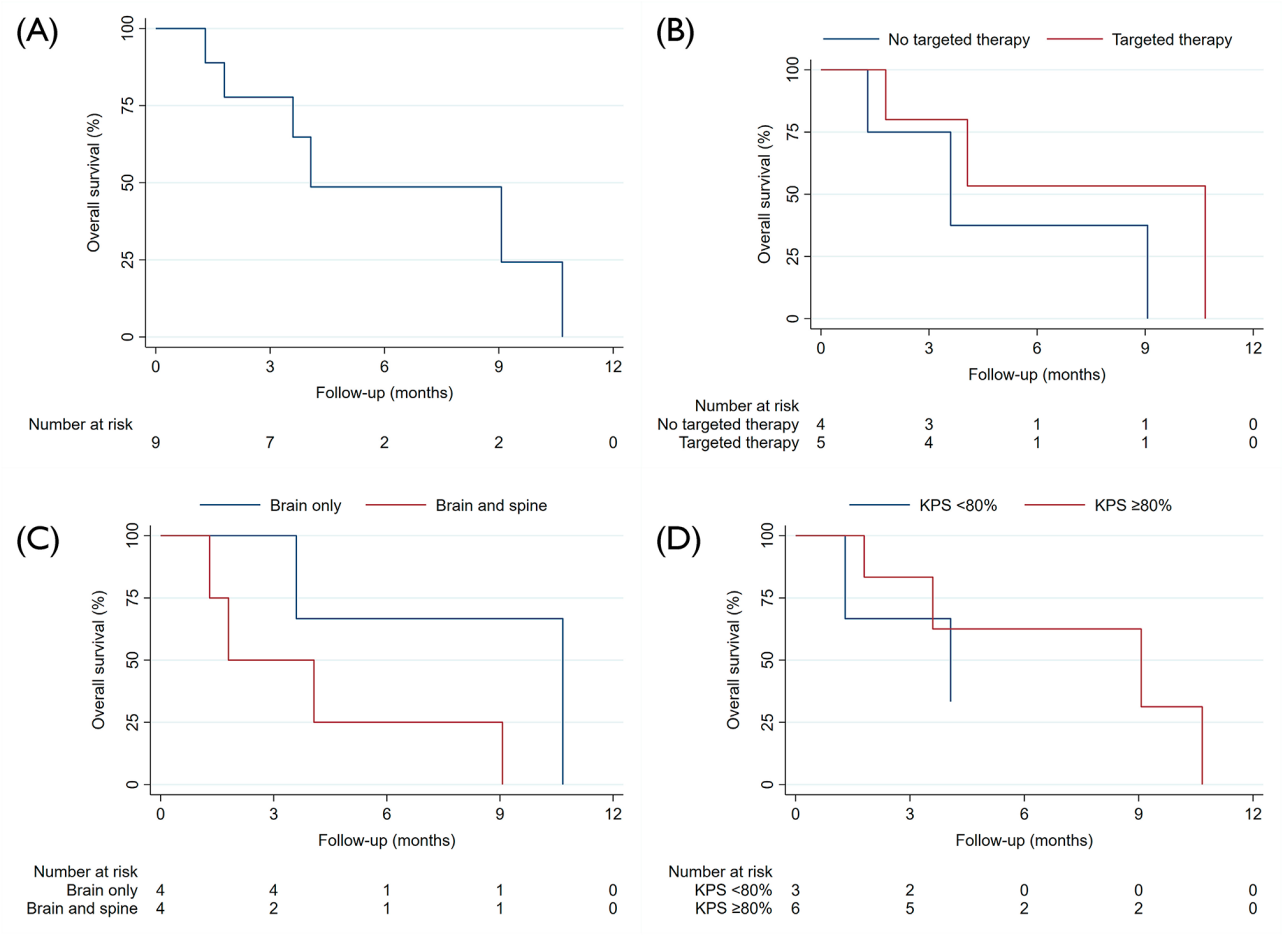
Overall survival for (**A**) Full cohort, (**B**) Stratified for concurrent targeted therapy, (**C**) Stratified for the extent of LMD, (**D**) Stratified for the median Karnofsky performance status

**Fig. 4:**
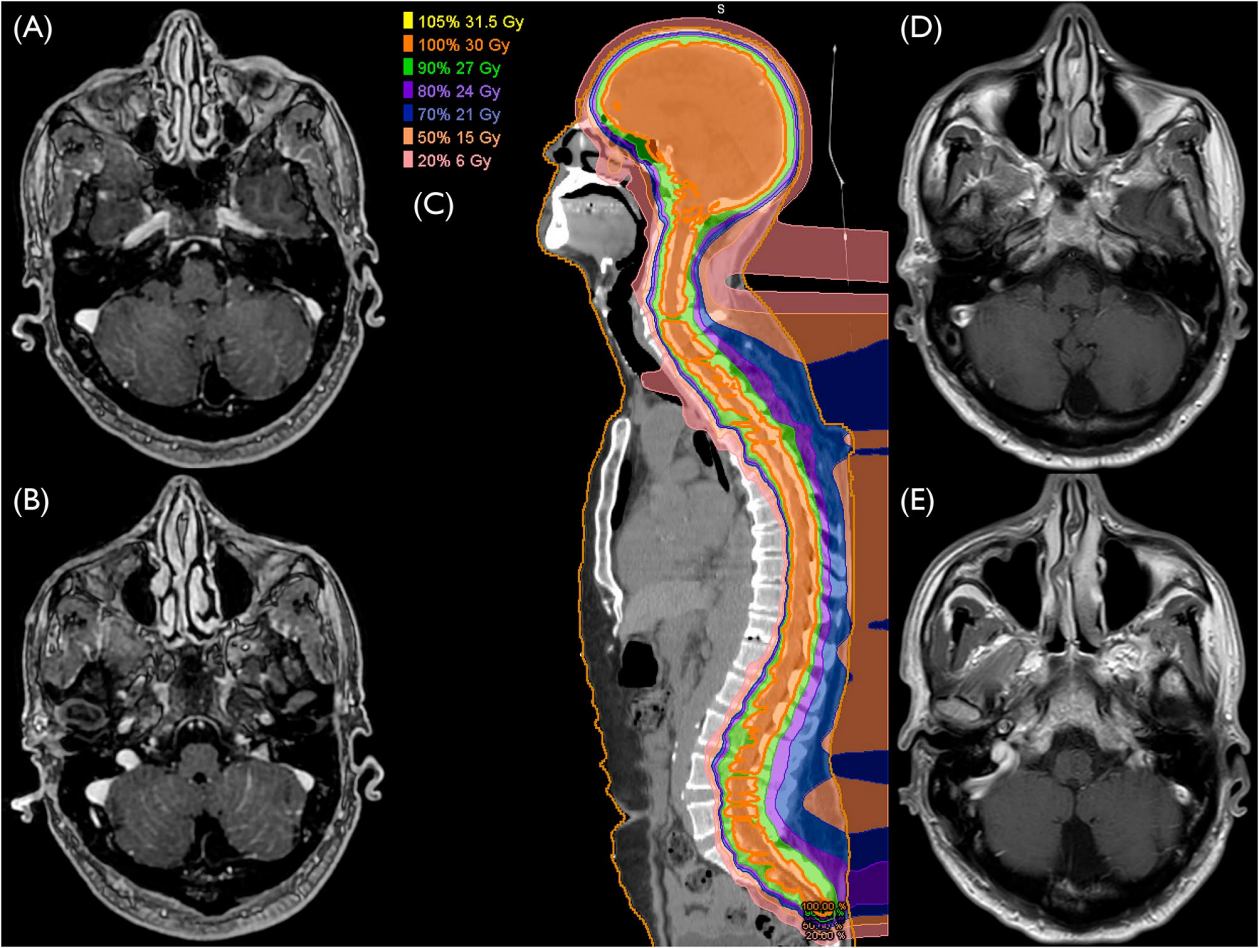
Case example. (**A**, **B**) 58-year-old male, KPS of 90%, with the diagnosis of extensive-stage small cell lung cancer and widespread cerebellar leptomeningeal disease on contrast-enhanced magnetic resonance imaging (MRI). He received chemotherapy with carboplatin and etoposide as well as immunotherapy with atezolizumab before and after proton craniospinal irradiation (pCSI) (**C**) pCSI treatment plan, 30 Gy (RBE) total dose, 10 fractions. (**D**, **E**) Three months after pCSI, contrast-enhanced MRI demonstrated complete resolution of LMD

The time from LMD diagnosis to the start of pCSI ranged from 30 to 84 days, with a median of 34 days. The median pCSI prescription dose was 30 Gy (RBE), administered in a consistent single fraction dose of 3 Gy across all patients. No patients received a simultaneously integrated boost, but one patient received a sequential boost of 6 Gy (RBE) in 3 fractions to the posterior fossa. The median duration of pCSI was 13 days, and all patients completed the treatment as planned. Seven patients (77.7%) were administered dexamethasone during pCSI. The median clinical and radiographic follow-up periods were 3.5 and 3.5 months, respectively. In three patients, CNS imaging after completion of pCSI was not available. The median time to CNS progression and CNS PFS were 7.5 months (95% CI: 2.7 – not available) and 4.0 months (95% CI: 1.3 – not available) (Fig. 1A and B). The median PFS for the full cohort was markedly shorter, with 2.7 months (95% CI: 0.7 – not available). (Fig. 2A). Meanwhile, the median OS for the patients was 4.0 months (95% CI: 1.3 – not available) (Fig. 3A). Six deaths and four disease progressions were observed. The median KPS 30 days after pCSI, available for six patients, was 75% (median split between 70 and 80%). Patients receiving targeted therapies demonstrated prolonged PFS and OS (Figs. 2B and 3B). Furthermore, younger patients (Supplementary File 1) and those with better KPS at pCSI were observed to have longer OS (Fig. 3D). However, patients with LMD located in both the brain and spine had shorter survival times than those who only had LMD in the brain (Figs. 2C and 3C). There were no meaningful differences in OS and PFS when comparing patients with active versus controlled extra-CNS disease or with or without concurrent immunotherapy or chemotherapy (data not shown). Patients with breast cancer and NSCLC had a prolonged PFS compared to those with other histologies, which did not translate into an improved OS (Supplementary File 2 and 3). The patient and treatment characteristics are summarized in Table 1. A patient case example is shown in Fig. 4.

**Table 1:** Patient, treatment, and toxicity data

Number of analyzed patients	9						
Sex (number of male/female)	2/7						
Treatment period	February 2023 to February 2024						
**Location of LMD** ^**†**^	**Brain only**		**Spine only**		**Brain and spine**		
Number of patients	4		0		4		
**Type of LMD**	**Linear**		**CSF-only**		**Linear and nodular**		
Number of patients	5		1		3		
**LMD diagnosis method**	**Imaging-only**		**CSF-only**		**Imaging and CSF**		
Number of patients	4		1		4		
**Tumor entity**	**NSCLC**	**SCLC**	**Breast**	**Esophageal**	**Gastric**	**Ovarian**	**Pancreatic**
Number of patients	1	1	3	1	1	1	1
**Presence of parenchymal brain metastases at time of pCSI**	**Yes**			**No**			
Number of patients	3			6			
**Extra-CNS disease status at pCSI**	**Active**			**Controlled**			
Number of patients	6			3			
**Concurrent chemotherapy**	**Yes**			**No**			
Number of patients	4			5			
**Concurrent targeted therapy**	**Yes**			**No**			
Number of patients	5			4			
**Concurrent immunotherapy**	**Yes**			**No**			
Number of patients	2			7			
	**Median**		**Mean (SD)**		**IQR**		**Range**
Age at start of pCSI (years)	58.6		58.3 (8.9)		51.8–67.2		45.4–70.9
Time from LMD diagnosis to pCSI start (days)	34		42.5 (18.7)		31–45		30–84
KPS before pCSI	80		76.6 (12.2)		70–80		50–90
Number of brain metastases at the time of pCSI	0		0.89 (1.9)		0–1		0–6
pCSI prescription dose (Gy RBE)	30		30.3 (1.0)		30–30		30–33
pCSI single fraction dose (Gy RBE)	3		3 (0)		3–3		3–3
Number of fractions	10		10.1 (0.3)		10–10		10–11
pCSI duration in days	13		14.1 (3.1)		13–14		11–21
KPS 30 days after pCSI	75		68.3 (16.0)		60–80		40–80
Clinical follow-up (months)	3.5		3.8 (3.4)		0.7–4.1		0.3–9.9
Radiographic follow-up (months)	3.5		5.2 (2.8)		3.4–7.7		3.4–9.9

**Toxicity** ^**‡**^	**Absolute number observed (%)**				
	**Grade 1**	**Grade 2**	**Grade 3**	**Grade 4**	**Grade 5**
Anemia	3 (50.0%)	2 (33.3%)	1 (16.6%)	0 (0%)	0 (0%)
Thrombocytopenia	2 (33.3%)	0 (0%)	0 (0%)	0 (0%)	0 (0%)
Leukopenia	2 (33.3%)	0 (0%)	1 (16.6%)	0 (0%)	0 (0%)
Fatigue	3 (50.0%)	2 (33.3%)	0 (0%)	0 (0%)	0 (0%)
Headache	2 (33.3%)	1 (16.6%)	1 (16.6%)	0 (0%)	0 (0%)
Nausea	0 (0%)	0 (0%)	2 (33.3%)	0 (0%)	0 (0%)
Weight Loss	2 (33.3%)	1 (16.6%)	1 (16.6%)	0 (0%)	0 (0%)
Generalized muscle weakness	2 (33.3%)	1 (16.6%)	1 (16.6%)	0 (0%)	0 (0%)
Alopecia	3 (50.0%)	0 (0%)	0 (0%)	0 (0%)	0 (0%)
Anorexia	2 (33.3%)	0 (0%)	1 (16.6%)	0 (0%)	0 (0%)
Blurred vision	0 (0%)	2 (33.3%)	0 (0%)	0 (0%)	0 (0%)

†: One patient with CSF-positive LMD alone without any macroscopic LMD

‡: Three patients lacked detailed clinical data on toxicity following the completion of pCSI

Abbreviations: LMD = leptomeningeal disease, CSF = cerebrospinal fluid, NSCLC = non-small cell lung cancer, SCLC = small cell lung cancer, pCSI = proton craniospinal irradiation, CNS = central nervous system, SD = standard deviation, IQR = interquartile range, RBE = relative biological effectiveness, KPS = Karnofsky Performance Status

Acute toxicity data were only available for six of the nine patients. Non-hematological grade 3 acute toxicities were observed in two patients (33.3%) (Table 1). One patient experienced a grade 3 headache and had to be admitted to the emergency department for a severe headache that was not relieved by pain medication. A shunt revision resolved the complaints. This patient was also admitted to the hospital on a separate occasion for grade 3 nausea and vomiting multiple times before collapsing from dehydration at home. The patient also suffered from grade 3 weight loss and anorexia due to taste changes, leading to inadequate oral intake. The patient experienced a 25% weight loss over 4 1/2 months, as documented around two months after pCSI completion. The nausea, weight loss, and anorexia resolved without medical intervention. Another patient tolerated pCSI poorly and developed grade 3 nausea, which caused them to complete treatment as an inpatient. After finishing radiation, she was generally weak and experienced an aspiration event, resulting in sepsis and requiring almost two months in the hospital and rehabilitation. Her KPS 30 days after pCSI was 40%. The patient also developed grade 3 generalized muscle weakness, requiring assistance to walk after treatment. Three patients experienced grade 2 toxicities, one patient for blurred vision, another for fatigue, and the last for fatigue, headache, weight loss, and blurred vision. Hematological grade 3 toxicity was observed in 2 patients (33.3%). The patient course of one of the affected patients has already been described. This patient had to undergo surgery for a shunt revision and developed leukopenia and multiple other issues, including nausea, vomiting, and weight loss. The other patient who suffered from ovarian cancer had a grade 3 anemia in the setting of a previously known anemia of chronic disease. In addition, both patients with hematological grade 3 toxicity received systemic treatments, including targeted therapies and chemotherapy, 60 days before, during, or 60 days after pCSI.

## Discussion

Herein, we report our first institutional series using pCSI in adult LMD patients. Within this early experience, there was a range of responses from poor to promising. Younger age, good performance status, and concurrent administration of targeted therapies showed favorable outcomes, suggesting a refined subgroup of patients may best benefit from pCSI. Patients with NSCLC or breast cancer only had a prolonged PFS without any superior OS. Conversely, patients suffering from LMD spread to both the spine and brain demonstrated poor outcomes. We observed a moderate rate of non-hematological acute grade 3 toxicity, affecting 33.3% of patients with available data. A similar rate was observed for hematological grade 3 toxicity. It is important to note that for the latter, patients received systemic treatments, experienced non-pCSI-related complications, or had pre-existing comorbidities, all of which could have affected their hematological function. Nevertheless, the toxicity profile of pCSI was manageable with no treatment-limiting toxicity. They were nonetheless clinically impactful on quality of life, especially considering the short survival after pCSI. These results generally agree with the available literature but fall short of the two recently published trials conducted at the Memorial Sloan Kettering Cancer Center (Table 2) [7, 8].

**Table 2:** Overview of available pCSI LMD data in adult patients

Author	Year	Study type	Number of patients	Age	Tumor entities	Only solid tumors?	Treatment modality	pCSI prescription dose and fractions	PFS	OS	Toxicity
Yang et al. [7]	2021	Prospective phase I	24 (21 eligible for analysis)	Median: 52 years Range: 30–67 years	NSCLC (*n* = 11), breast carcinoma (*n* = 7), esophageal adenocarcinoma (*n* = 1), rectal adenocarcinoma (*n* = 1), adenoid cystic carcinoma of parotid (*n* = 1)	Yes	pCSI	30 Gy in 10 fractions (RBE)	Median CNS PFS: 7 months, 95% CI: 5–13	Median: 8 months, 95% CI: 6 - not reached	20 patients eligible for analysis: Fatigue [19 patients (95%)]: grade 1 (*n* = 10, 50%), grade 2 (*n* = 8, 40%), grade 3 (*n* = 1, 5%) Lymphopenia [18 patients (90%)]: grade 2 (*n* = 1, 5%), grade 3 (*n* = 15, 75%), grade 4 (*n* = 2, 10%) Thrombocytopenia [10 patients (50%)]: grade 1 (*n* = 7, 35%), grade 2 (*n* = 2, 10%), grade 4 (*n* = 1, 5%)
Yang et al. [8]	2022	Prospective randomized phase II	98 (42 pCSI, 21 IFRT, 35 exploratory pCSI)	Median: 57 years (pCSI), 61 years (IFRT), 61 years (exploratory pCSI) Range: 27–79 years	NSCLC (*n* = 36) and breast (*n* = 27), (other entities in the exploratory pCSI group: ovarian (*n* = 7), esophageal (*n* = 6), melanoma (*n* = 6), colorectal (*n* = 5), head and neck (*n* = 3), pancreatic (*n* = 2), SCLC (*n* = 1), anal (*n* = 1), biliary (*n* = 1), prostate (*n* = 1), unknown primary (*n* = 1))	Yes	pCSI vs. photon IFRT	3 Gy in 10 fractions (RBE)	Median CNS PFS: 7.5 months (pCSI), 2.3 months (IFRT)	Median: 9.9 months (pCSI), 6.0 months (IFRT)	pCSI grade 4: lymphopenia (10%) pCSI grade 3: fatigue (2%), pain (2%), vomiting (2%) No difference between pCSI and IFRT
Lam et al. [9]	2024	Retrospective	45	Median: 54 years Range: 23–79 years	Breast cancer (*n* = 24), NSCLC (*n* = 8), melanoma (*n* = 4), gastrointestinal cancer (*n* = 2), atypical neuroendocrine lung cancer (*n* = 1), other (*n* = 6)	Yes	pCSI	3 Gy in 10 fractions (RBE) (*n* = 39)	Median PFS: 6.5 months	Median: 13.7 months	Non-hematologic events during or right after pCSI: 76% of patients had nausea, 51% headache, 31% fatigue, 4% dizziness Hematologic events: overall numerical decrease in white blood count, hemoglobin level, and platelet count 4 weeks after radiation, recovered by 8 weeks after pCSI, no hematologic values were critically low
Webb et al. [10]	2023	Retrospective	1	60 years	Large cell neuroendocrine carcinoma (*n* = 1)	Yes	pCSI, bevacizumab, and pembrolizumab	30 Gy in 10 fractions (RBE not specified)	4.6 months	6 months	ICI-induced hypophysitis, lymphopenia, thrombocytopenia, and fatigue
Sener et al. [11]	2024	Retrospective	2	Not specified (one patient “in his 30s”, one patient “in his 70s”)	Melanoma (*n* = 2)	Yes	pCSI and various ICI	30 Gy in 10 fractions (RBE not specified)	Patient 1: 3 months; Patient 2: 5 months	Patient 1: 7 months (still alive); Patient 2: 5 months (still alive)	Acute: grade 1 fatigue, grade 1 headache; Late: ICI-induced hepatitis

Abbreviations: pCSI = proton craniospinal irradiation, IFRT = photon involved-field radiotherapy, NSCLC = non-small cell lung cancer, SCLC = small cell lung cancer, ICI = immune checkpoint inhibitor, RBE = Relative biological effectiveness

The first of these two studies is a phase I trial that examined the outcomes of 24 patients with LMD from solid tumors that were treated with pCSI (30 Gy RBE in 10 fractions) [7]. Twenty patients were evaluable for treatment-related toxicities, with 21 patients assessable for CNS PFS and OS [7]. Only two patients experienced dose-limiting toxicities, including lymphopenia, thrombocytopenia, and fatigue, all of which resolved without medical intervention. Despite the diverse patient population, the median OS was 8 months, and the median CNS PFS was 7 months. Notably, four patients achieved CNS disease control for over a year. Moreover, patient-reported symptom assessment using the MD Anderson Symptom Inventory – Brain (MDASI-BT) and MDASI ‒ Spine mainly demonstrated posttreatment symptom stability among the tested domains.

These promising results led to a randomized phase II trial comparing pCSI with photon IFRT [8]. In this trial, 63 patients with LMD from breast cancer or NSCLC were treated. The patients were randomized in a 2:1 fashion, favoring the pCSI arm (30 Gy RBE in 10 fractions). At the planned interim analysis, CNS PFS and OS were markedly prolonged in patients receiving pCSI. The median CNS PFS in the pCSI group was 7.5 months, compared with 2.3 months in the control arm (IFRT). A comparable advantage was observed for OS (9.9 versus 6.0 months). There was no significant difference in the rate of serious adverse effects between the pCSI and IFRT. In addition, an exploratory cohort of 35 patients with other histologies treated with pCSI was analyzed. The exploratory cohort mainly comprised ovarian (*n* = 7) and esophageal cancer (*n* = 6), as well as malignant melanoma (*n* = 6). The median CNS PFS and OS were 5.8 and 6.6 months, and, therefore, shorter than the breast cancer and NSCLC cohort.

We did not observe meaningful differences in OS when comparing patients with breast cancer and NSCLC to those with other histologies (data not shown) but were limited by the small number of patients. CNS disease control and OS with pCSI might remain limited in selected patients, and LMD progression continues to be a challenge. Additional therapies may be necessary to enhance the efficacy of pCSI and improve treatment outcomes. Nevertheless, the encouraging results of the discussed phase I and II trials have led to the development and approval of NRG-BN014, a phase 3 randomized trial comparing pCSI and IFRT for patients with LMD from breast cancer or NSCLC (NCT06500481).

The blood-brain barrier poses a significant challenge in treating brain metastases and LMD by preventing many therapeutic agents from fully entering the CNS [13]. Compared to earlier EGFR-tyrosine kinase inhibitors (TKIs), osimertinib demonstrates an increased ability to cross the blood-brain barrier [14]. The phase I BLOOM study investigated the role of osimertinib in EGFR-mutant NSCLC with patients with leptomeningeal metastasis who failed on previous EGFR TKI [15]. A total of 41 patients were enrolled. The median PFS and OS were 8.6 and 11.0 months, respectively, underlining the promising role of EGFR-directed treatments in LMD patients. While one of our patients received osimertinib shortly before pCSI, others were treated with sacituzumab govitecan (breast cancer), mirvetuximab (ovarian cancer), and abemaciclib (breast cancer). Acknowledging the cohort heterogeneity and number of analyzed patients, the tendency of longer PFS and OS suggests the potential benefit of targeted drugs. The role of combining targeted treatments with pCSI to achieve synergistic effects to improve outcomes further calls for more research.

Given the central role of immunotherapy in many advanced malignancies, its efficacy in LMD is also of particular interest. A retrospective case study with two patients explored the feasibility and potential benefits of combining pCSI with immune checkpoint inhibitors (ICI) in treating LMD from malignant melanoma [11]. Patient 1, a male in his 30s with BRAF V600E-mutant melanoma, was initially treated with ipilimumab and nivolumab upon diagnosis of widespread metastatic disease. After developing ICI-induced hepatitis, his treatment was changed to encorafenib and binimetinib. Following a LMD diagnosis shortly after, he received pCSI, 30 Gy in 10 fractions, alongside nivolumab and later a combination of dabrafenib, trametinib, and relatlimab. Despite CNS progression, he remained alive 7 months post-LMD diagnosis, demonstrating the feasibility and potential benefit of this treatment approach. Patient 2, a male in his 70s with BRAF-wildtype melanoma, received multiple treatments, including stereotactic radiosurgery for a left frontal brain metastasis and pembrolizumab, over several years. Upon LMD diagnosis, roughly 8 1/2 years after initial diagnosis, he was treated with pCSI, 30 Gy in 10 fractions, combined with ipilimumab and nivolumab. He remained progression-free and asymptomatic for 5 months post-LMD diagnosis, with resolution of leptomeningeal enhancement observed on imaging. The study demonstrated that pCSI combined with ICI is feasible and can be tolerated by patients with manageable toxicity.

These results are comparable to what we observed from a 58-year-old male with a diagnosis of extensive-stage small cell lung cancer and widespread cerebellar LMD (Fig. 4). He underwent pCSI while also receiving atezolizumab. Post-pCSI, the patient experienced no acute grade 3 toxicities, reflecting a good tolerance to the treatment. Furthermore, follow-up imaging conducted three months after the completion of pCSI revealed an excellent response to the treatment. There was a complete interval resolution of previously observed widespread leptomeningeal enhancement throughout the cerebellar folds and over both cerebral convexities, along with no evidence of metastatic disease in the brain. These case studies suggest the need to validate and optimize the combination of immunotherapy and pCSI as a valid treatment approach.

Another case report discussed a 60-year-old male patient with LMD from large cell neuroendocrine carcinoma who was treated with pCSI, bevacizumab, and pembrolizumab [10]. This patient had a PFS of 4.6 months and OS of 6.0 months, comparable to the median CNS PFS and OS of our cohort, which were both 4 months. However, adding bevacizumab and pembrolizumab in the case report seemed to provide a slightly longer OS of 6.0 months and improved toxicity outcomes, albeit with the risk of ICI-induced toxicities. The treatment regimens in both studies involved similar pCSI doses and durations, but this case report highlights the potential for combination therapy to enhance clinical benefits.

The recent Society for Neuro-Oncology (SNO) and American Society of Clinical Oncology (ASCO) consensus review highlighted the recent advances in the understanding and management of LMD [3]. Yet, with selected LMD patients demonstrating considerable survival times compared to historical cohorts, further research is necessary to refine treatment algorithms and patient selection. Given the central role of radiation therapy in treating pCSI, the results after pCSI are encouraging but underline the need to identify patients benefitting the most (see NRG-BN014). In addition, the growing but still limited access and considerable costs of pCSI remain significant barriers to the widespread implementation of this treatment option [16]. However, as many previous works on photon-based craniospinal irradiation underlined the considerable risk of high-grade toxicity, the significant advances in treatment techniques have to be acknowledged. For instance, photon volumetric modulated arc therapy (VMAT) with vertebral body sparing appears to be a potential alternative with tolerable toxicity [17]. A recent study of ten adult patients with LMD from solid tumors demonstrated a favorable safety profile after photon VMAT craniospinal irradiation [17]. Notably, no patient experienced any grade 3 toxicity, with a total of 13 grade 2 toxicities. While current studies on this topic are limited due to their small sample size and cohort heterogeneity, further research is warranted to characterize the role of photon-based state-of-the-art treatment techniques for craniospinal irradiation.

There are several limitations to this study that must be considered when interpreting the reported findings. The retrospective nature of the analysis limits the ability to establish causality, and the single-center design may not represent broader patient populations or different clinical settings. Additionally, the small sample size restricts the interpretability, limiting the detection of meaningful and subtle differences or trends in efficacy, survival outcomes, or patient-reported symptoms. Changes in patient-reported symptoms are confounded by multiple clinical factors, including changes in therapy and disease status, and should be interpreted cautiously.

## Conclusion

Our first institutional experience with pCSI in adult LMD patients suggests a potential role of pCSI for a well-defined patient subset. While the observed PFS and OS rates in LMD remain dismal, pCSI may offer a useful treatment option in carefully selected patients. Further research is warranted to identify these patients and refine existing treatment algorithms. Finally, the efficacy and safety of combination treatments with pCSI and targeted therapy or immunotherapy must be defined.

## Electronic supplementary material

Below is the link to the electronic supplementary material.

Supplementary Material 1

Supplementary Material 2

Supplementary Material 3

## Data Availability

The data analyzed in this study are available from the corresponding author upon reasonable request.
