# Supplementary figures and images for: Early experience with proton craniospinal irradiation in adult patients with leptomeningeal disease
Source: Radiat Oncol. 2025 Apr 22;20:61. doi: 10.1186/s13014-025-02618-7 (PMC12016269; doi:10.1186/s13014-025-02618-7)

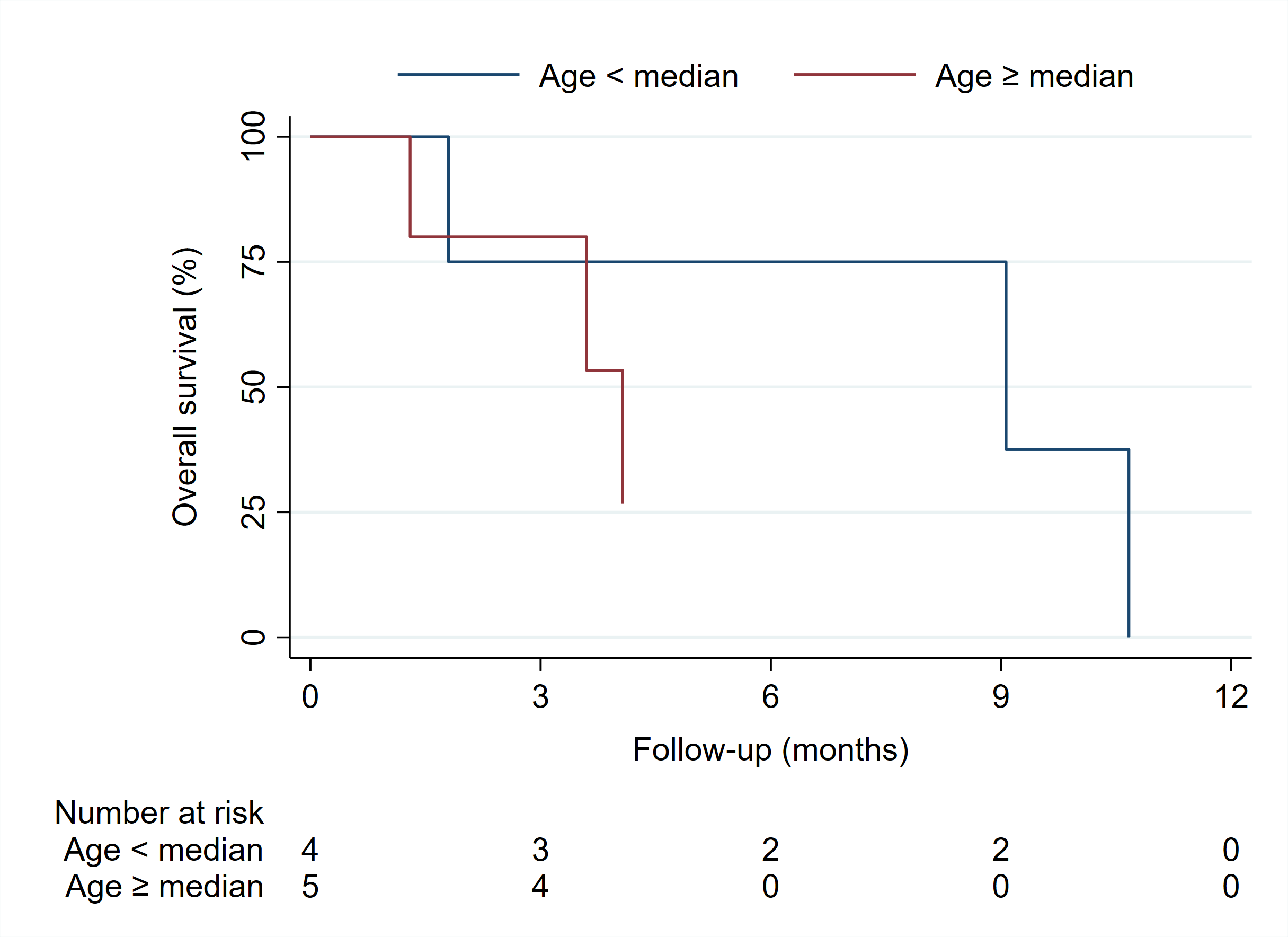

Supplement: Supplementary file 1 — Supplementary Material 1 [file 13014_2025_2618_MOESM1_ESM.png]

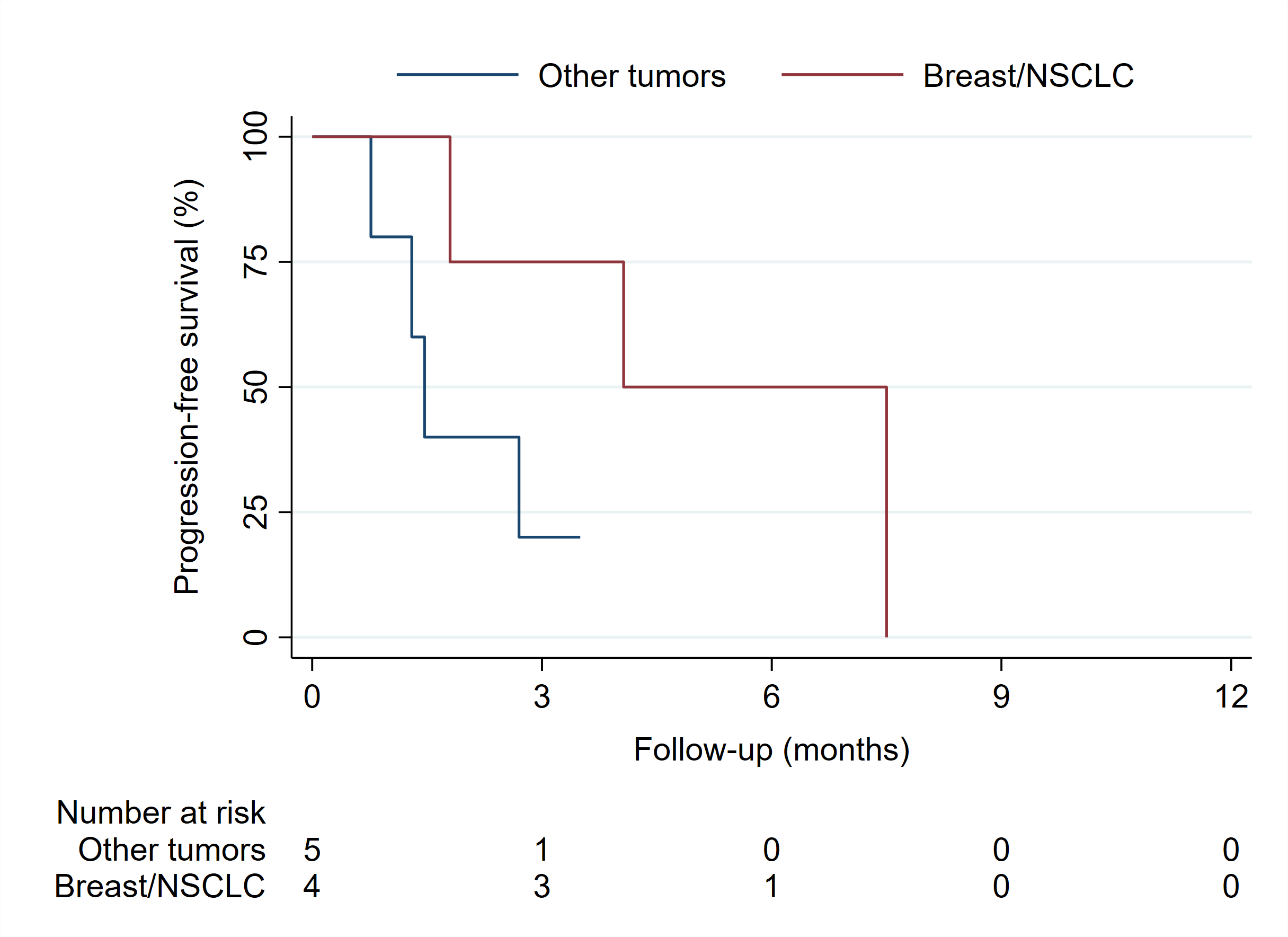

Supplement: Supplementary file 2 — Supplementary Material 2 [file 13014_2025_2618_MOESM2_ESM.png]

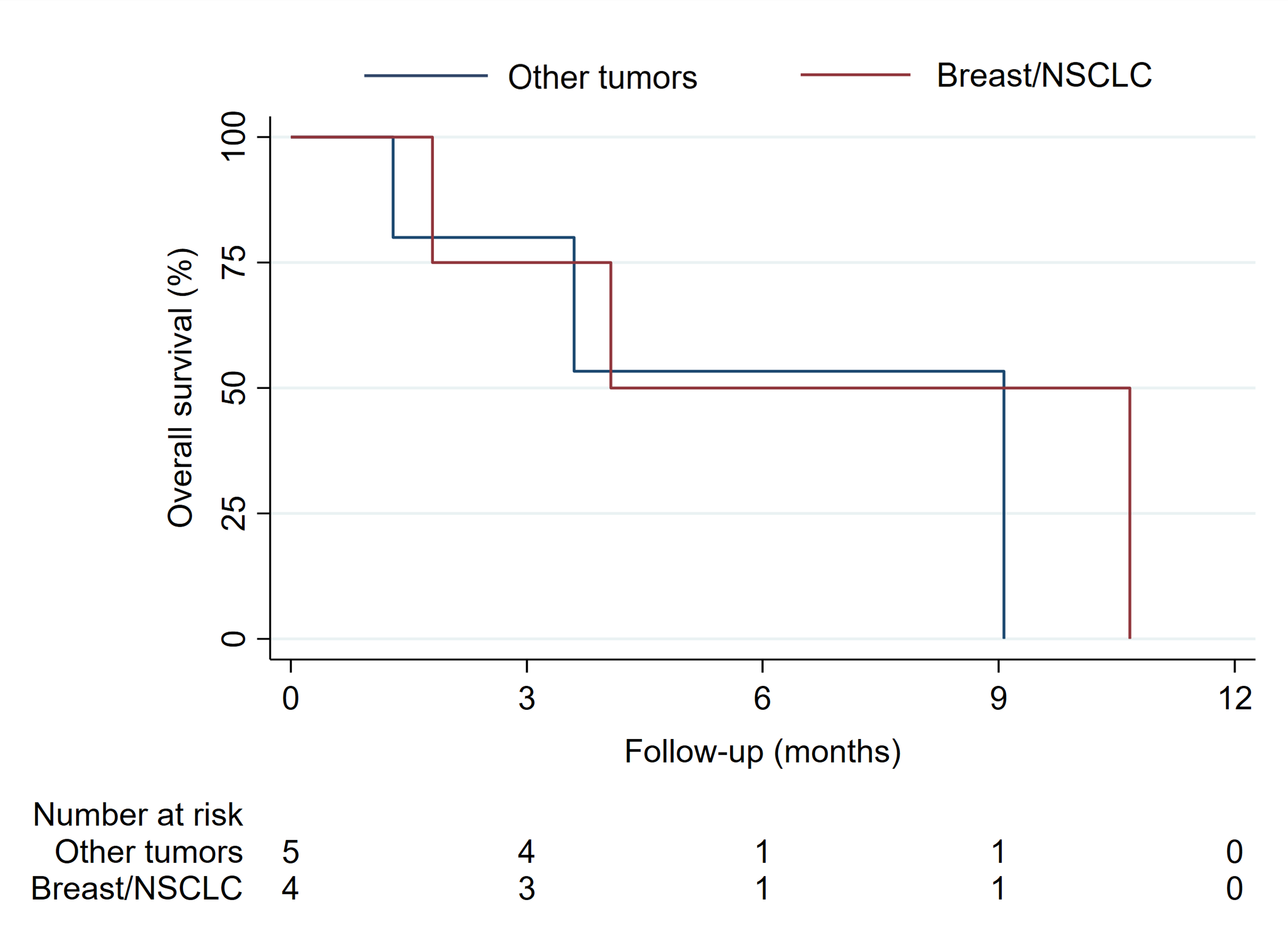

Supplement: Supplementary file 3 — Supplementary Material 3 [file 13014_2025_2618_MOESM3_ESM.png]
